# Marital status and suicidal behavior in South Asia: A systematic review and meta‐analysis

**DOI:** 10.1002/hsr2.1781

**Published:** 2023-12-20

**Authors:** S. M. Yasir Arafat, Vikas Menon, Md Abdullah Saeed Khan, Mila Nu Nu Htay, Rakesh Singh, Deepika Biyyala, Yuvaraj Krishnamoorthy, Keerthana Mynampally

**Affiliations:** ^1^ Department of Psychiatry Enam Medical College and Hospital Dhaka Bangladesh; ^2^ Department of Psychiatry Jawaharlal Institute of Postgraduate Medical Education and Research Puducherry India; ^3^ Department of Community Medicine National Institute of Preventive and Social Medicine (NIPSOM) Dhaka Bangladesh; ^4^ Department of Community Medicine Manipal University College Malaysia, Faculty of Medicine Melaka Malaysia; ^5^ Department of Research – Transcultural Psychosocial Organization Nepal Kathmandu Nepal; ^6^ Department of Psychiatry AIIMS Jodhpur India; ^7^ Department of Community Medicine ESIC Medical College & Hospital Chennai India

**Keywords:** married, South Asia, suicidal behavior, suicide, suicide attempt

## Abstract

**Background and Aims:**

The connection between marital status and suicidal behavior has been poorly assessed in South Asia. We aimed to see the proportion of marital status in individuals with suicidal behavior in South Asian countries.

**Methods:**

We followed PRISMA guidelines and registered the protocol in advance (PROSPERO 2023 CRD42023399906). A systematic search was conducted in Medline, Embase, and PsycINFO. Meta‐analyses were performed to pool the proportion of married individuals with suicidal behavior (total [suicide + suicide attempts], suicide, and suicide attempt) in South Asian countries. We considered suicidal behavior consist of suicide and suicide attempts (nonfatal).

**Results:**

Our search identified 47 studies for this review from 6 countries published from 1999 to 2022 with a sample size ranging from 27 to 89,178. The proportion of married individuals was 55.4% (95% CI: 50.1–60.5) for suicidal behavior, 52.7% (95% CI: 44.5–60.7) for suicides, and 43.1 (95% CI: 32.9–53.9) for suicide attempts. The proportion of married persons among suicide attempts varied significantly across countries (*p* = 0.016) which was highest (61.8%; 95% CI: 57.2–66.2) in India, followed by Bangladesh (52.5%; 95% CI: 41.8%–62.9%) and Pakistan (45.1%; 95% CI: 30.9–59.9). The pooled proportions did not differ significantly in relation to the quality of the studies (*p* = 0.633).

**Conclusion:**

This review identified married persons died more than others by suicide in South Asian countries while single persons attempted suicide than married. As the current study did not assess any cause‐and‐effect association, a cautious interpretation is warranted while considering married marital status as a risk factor.

## 1 INTRODUCTION

Suicide having a linkage with various factors is considered a serious public health issue. Worldwide, in 2019, around 703,000 people lost their life by suicide.^1^ More than three‐quarters of this loss occurred in low‐ and middle‐income countries,^1^ indicating the necessity of urgent attention to decrease suicidal behavior.

Suicide is the end product of a network of interactions among multiple risk factors,^2^ such as biological factors, psychological factors, social structure, ecological factors, cultural factors, and religious factors. Several theories explaining the multiple interactions and attributing role of any individual variable have been discussed. However, any specific risk factor for suicidal behavior is yet to be revealed. Despite mental health being one of the major risk factors for suicide, a systematic review found that psychiatric disorders had a similar population‐attributable risk for suicide in terms of socioeconomic factors,^3^ warranting the significance of social factors for improving population health and reducing the burden of suicide. Moreover, the odds of suicide are higher during periods of socioeconomic, family, or individual crisis.^2^

Among various psychosocial factors, marital status is linked with social and community integration,^4^ and in turn, is associated with social isolation and its further consequences including suicidal behavior.^5^ While marriage could enhance social integration and regulation leading to chances of reducing suicidal risk, divorce, on the other hand, could increase suicide risk by breaking the marriage and relationships between the individual.^4^ Several studies have examined to demonstrate that marital status is a significant factor in suicide and have found that single people are significantly more likely to die by suicide than married people.^6^, ^7^, ^8^, ^9^, ^10^, ^11^, ^12^ Similarly, cultural and geographical factors are also related to developing suicidal behavior. For example, marriage acting as a protective factor is subject to culture‐specific.^7^ Likewise, the sociocultural and economic contexts of Asian nations differ from Western nations when it comes to suicide.^13^, ^14^, ^15^, ^16^ Age of marriage could be an important attributing factor for this variation as marriage occurs relatively in older ages in Western countries due to different legal structures of divorce and property.

South Asia (Afghanistan, Bangladesh, Bhutan, India, Maldives, Nepal, Pakistan, and Sri Lanka) is home to one‐fifth of all mental health cases and accounts for approximately 25% of the global population.^17^ About one quarter of global suicides were happened in India in 2019.^1^ Additionally, among the eight countries, India, Nepal, Pakistan, and Sri Lanka had suicide rates more than the global average. However, there is a dearth of studies assessing the association of suicidal behavior and sociocultural and ecological factors like marital status in South Asia, a region with a high rate of suicide. As there is scattered evidence on suicide and marital studies in South Asian countries, we attempt to conduct a meta‐analysis after a systematic review by looking at published (i.e., peer‐reviewed) studies conducted in South Asian countries. As a result, we aimed to assess the proportion of marital status of individuals with suicidal behavior in South Asian countries.

## 2 METHODS

### 2.1 Search strategy

We made a systematic search in three databases (Medline, Embase, and PsycINFO) by predesigned search terms to identify available papers. We also performed hand search in previously published reviews.^17^, ^18^, ^19^ The search details are mention in Supporting Information: File 1 and the review protocol was registered in advance (PROSPERO 2023 CRD42023399906). We searched the data bases from inception to search date (February 4, 2023).

### 2.2 Inclusion criteria

We included original research contributions, studies with quantitative estimates, published in the English language, and articles available in full‐text were included. The population included in this review was restricted to studies in South Asian countries (Afghanistan, Bangladesh, Bhutan, India, Maldives, Nepal, Pakistan, and Sri Lanka) in humans. Only studies mentioning the marital status of persons with suicidal behavior, that is, suicide, suicide attempt, or both (fatal and/or nonfatal suicide attempts irrespective of suicidal intent) were included.

### 2.3 Exclusion criteria

We excluded articles discussing suicidal behavior among veterans, and articles with qualitative outcomes. We also excluded any type of review, editorial, erratum, letters without primary data, and multiple articles from same projects. In cases of multiple papers from a single project, we included the paper providing the data in maximum extent regarding marital status and suicidal behavior.

### 2.4 Study selection

**Figure 1:**
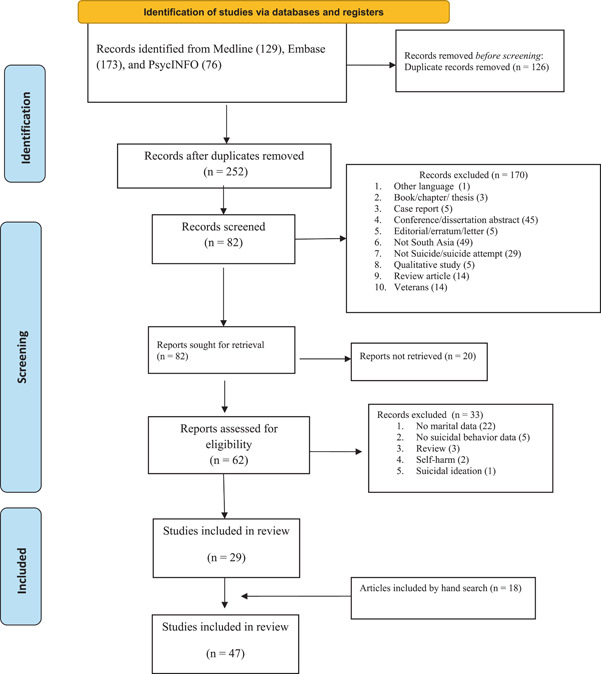
PRISMA 2020 flow diagram.

The studies were independently screened by two review authors (S. M. Y. A. and V. M.) and a third review author (R. S.) was consulted when needed. We followed PRISMA flow chart and mentioned the stepwise details of the search in Figure 1.

### 2.5 Data extraction

We extracted the study details (name of the lead author, year of publication, name of the journal), country where the study was conducted, place where the study was conducted, instruments measuring suicidal behavior, duration of the study, data collection year, study design, data collection methods, study setting (rural/urban), sample size, male–female ratio (when applicable), type of suicidal behavior (attempt/suicide/both), and marital status. We considered the marital status in two groups (married and others [never married/unmarried, separated, widow/widower, divorced]). We created these two groups because there were small proportions of divorced, widow/widower, and separated persons. Also, we could not exclude them from the analysis to maintain the sample size of the study. Data were extracted by two review authors (D. B. and K. M.) independently in Microsoft Excel version 10 and a third review author was involved (R. S.) when necessary and checking.

### 2.6 Quality assessment

Among the included articles, the cross‐sectional studies' methodological quality as assessed by using Newcastle Ottawa Scale (NOS) that was adapted for the cross‐sectional studies.^20^ The methodological quality of the case control studies was assessed by using Newcastle Ottawa Quality Assessment Scale for case control studies.^21^ Two authors (M. N. N. H. and S. M. Y. A.) independently assessed the risk of bias of included studies. For cross‐sectional studies, the NOS scale is assessed on three domains: (1) sample selection, (2) comparability of the different outcome groups, and (3) outcome assessments and statistical analysis. While for case control studies, (1) selection of cases and controls, (2) comparability, and (3) exposure domains were assessed for methodological quality. In both scales, the total score was summed up and evaluated as low risk of bias (7 and above), moderate risk of bias (4–6) and high risk of bias (3 and below).

### 2.7 Data analysis

RStudio (version 2023.06.0+421) and statistical package meta were used for meta‐analysis. The proportion of married individuals (with 95% confidence interval [CI]) in total suicidal behavior, suicide attempts, and suicide was pooled using both fixed and random effects models. The heterogeneity among studies was explored using both the Cochran's *Q* and the *I* ^2^ statistic. Subgroup analysis was carried out across type of suicidal behavior (fatal and nonfatal), country (i.e., Bangladesh, India, and Pakistan), and study quality (low, moderate, and high). Groups with less than three studies were omitted from the subgroup analysis to avoid distorted and nongeneralizable estimates. The random effect estimates were used because of high heterogeneity among studies. A prediction interval was also estimated to provide a range of expected prevalence of married individuals among suicide cases. Publication bias was not assessed as the assumption that positive results are preferentially published is not necessarily true for proportional studies.^22^

### 2.8 Ethical aspects

We did not seek institutional review board approval for this review as we reviewed publicly available articles.

## 3 RESULTS

### 3.1 Characteristics of included studies

Our search identified 47 studies for this review from 6 countries (Bangladesh [8], India [27], Nepal [1], Pakistan [9], and Sri Lanka [2]) (Table 1). We did not find any studies from Bhutan and the Maldives. Studies were published between 1999 and 2022 (Table 1). Suicide was the outcome variable in 30 studies, suicide attempt was found in 8 studies, and the rest of the studies include suicidal behavior (suicide and suicide attempt). Sample size ranges from 27 to 89,178, 23 studies were conducted in urban settings, 7 were in rural areas, and the 17 studies had mixed samples from both urban and rural areas. Data were collected by interview in 32 studies and different records were reviewed in the rest studies.

**Table 1:** Characteristics of studies (*n* = 47).

S. no.	Study	Country	Place of study	Study duration (month)	Data collection year	Data collection methods	Study setting	Sources of cases	Suicidal behavior	Method of suicidal behavior	Number of cases	Male	Female	Age of respondents (years) mean (SD)
1	Abdullah et al.^23^	Pakistan	Khyber Pakhtunkhwa	8	2015	Psychological autopsy interviews	Urban	Hospital	Fatal	Mixed	63	38	25	22.1 + 3.1
2	Acherjya et al.^24^	Bangladesh	Jashore	6	2018	Interview	Urban	Hospital	Fatal	Poisoning	474	223	251	27 ± 11
3	Ahmad et al.^25^	Pakistan	Karachi	60	2011–2015	Record review and interviews	Urban	Police records and poison center	Both	Mixed	700	450	250	28.2 ± 8.8 in male, 26.1 ± 8.3 in female
4	Ali et al.^26^	Pakistan	Punjab	48	2018–2021	Interview	Urban	Community	Fatal	Mixed	100	60	40	26
5	Ambade et al.^27^	India	Maharashtra	36	1998–2000	Record review	Urban	Mortuary data and police records	Fatal	Mixed	1127	704	423	
6	Ambade et al.^28^	India	Maharashtra	60	2001–2005	Record review	Rural	Police and autopsy records	Fatal	Hanging	127	107	20	10–80
7	Arafat et al.^29^	Bangladesh		12	2018–2019	Reviewing online news reports	Both	Community	Both	Mixed	199	94	105	26.9 ± 13.6
8	Arafat et al.^30^	Bangladesh		12	2018–2019	Reviewing of print news reports	Both	Community	Both	Mixed	403	179	224	25.8 ± 11.6
9	Arafat et al.^31^	Bangladesh	Dhaka	13	2019–2020	Interviews	Urban	Community	Fatal	Mixed	100	49	51	26.3 ± 12.4
10	Arafat et al.^32^	Bangladesh		120	2009–2018	Reviewing online news content	Both	Community	Both	Mixed	358	142	215	23.8 ± 11.4
11	Armstrong et al.^33^	India	Tamil Nadu	7	2016	Reviewing print news papers	Both	Community	Fatal	Mixed	988	467	521	
12	Badiye et al.^34^	India	Maharastra	60	2009–2013	Record review	Urban	Records from crime branch	Fatal	Mixed	2306	1647	659	
13	Bansal et al.^35^	India	Punjab	12	2010	Interview	Urban	Hospital	Nonfatal	Mixed	100	61	39	26.9 ± 8.1
14	Bashir et al.^36^	Pakistan	Karachi	6		Interview	Urban	Hospital	Nonfatal	Poisoning	374	230	144	25 ± 10.1
15	Bastia and Kar^37^	India	Cuttack	24	1998–1999	Interview and record review	Urban	Community	Fatal	Hanging	104	43	61	28.7 ± 11.4
16	Bhatia et al.^38^	India	Delhi	60		Reviewing suicide notes and interviews	Urban	Forensic data	Fatal	Mixed	40	26	14	
17	Bhatia et al.^39^	India	Delhi			Record review, interviews	Urban	Hospital	Both	Mixed	373	189	184	
18	Bhise and Behere^40^	India	Maharashtra	18	2008–2009	Interview	Rural	Community people	Fatal	Mixed	98	88	10	
19	Chandrasekaran and Gnanaselane^41^	India	Puducherry	12	2001–2002	Interview	Mixed	Hospital	Nonfatal	Mixed	341	153	188	26.1 ± 9.3
20	Chaudhari et al.^42^	India	Puducherry	60	2010–2014	Record review	Both	Forensic records	Fatal	Poisoning	595	363	232	35.8 + 14.6
21	Fernando et al.^43^	Sri Lanka	Colombo	12	2006	Interview	Urban	Court records	Fatal	Mixed	151	93	58	
22	Hagaman et al.^44^	Nepal	Nepal	4	2015–2016	Interview and reviewing police records	Both	Community	Fatal	Mixed	302	172	130	32.9 + 17.5
23	Halder and Mahato^45^	India	Kolkata	24	2013–2014	Interview	Urban	Hospital	Nonfatal	Mixed	100	28	72	23.5 ± 6.4
24	Kar^46^	India	Orissa	24	1994–1996	Interview	Urban	Hospital	Nonfatal	Mixed	149	65	84	31.6 ± 13.5
25	Khan et al.^47^	India	Secunderabad	1	2005	Interview	Both	Hospital	Fatal	Mixed	50	29	21	
26	Khan et al.^48^	Pakistan	Karachi	12	2003	Interview, psychological autopsy method	Urban	Community people	Fatal	Mixed	100	83	17	
27	Khan et al.^49^	Pakistan	Ghizer	48	2000–2004	Police records and Interview	Urban	Police records	Fatal	Mixed	49		49	
28	Kumar et al.^50^	India	Lucknow	60	2008–2012	Record review	Both	Hospital	Fatal	Burning	857	66	791	33.7 ± 11.6
29	Kumar and Hashim^51^	India	Karnataka	36	2013–2015	Record review	Rural	Hospital	Fatal	Mixed	426	355	71	34.7
30	Kumar et al.^52^	India	Kerala	6	2004	Interview	Rural	Community	Fatal	Mixed	166	124	42	40.5 + 17.1
31	Manoranjitham et al.^53^	India	Tamil Nadu	20	2006–2008	Psychological autopsy interview	Rural	Community	Fatal	Mixed	100	59	41	42.2 ± 20.7
32	Mayer and Ziaian^54^	India			1995	Record review	Both	Community sample	Fatal	Mixed	89,178	52,357	36,821	
33	Mohanty et al.^55^	India	Berhampur	48	2000–2003	Record review, interviews	Both	Hospital	Fatal	Mixed	588	300	288	
34	Naz^56^	Pakistan	Punjab	10	2014–2015	Reviewing newspaper content	Both	Community people	Fatal	Mixed	87	50	37	
35	Pal et al.^57^	India	Madhya Pradesh	12	2020–2021	Interview	Urban	Hospital	Nonfatal	Mixed	60	38	22	39.03 ± 11.6
36	Parkar et al.^58^	India	Mumbai	84	1997–2003	Interview	Urban slums	Community people	Fatal	Mixed	76	33	43	
37	Patel et al.^59^	India		36	2001–2003	Interview	Both	Community sample	Fatal	Mixed	2684	1393	964	
38	Reza et al.^60^	Bangladesh		24		Interview	Rural	Hospital	Both	Mixed	113	44	69	29.6 ± 12.8
39	Sadia et al.^61^	Pakistan	Sargodha	12	2019	Record review	Both	Hospital	Both	Wheatbill (aluminum phosphide)	83	42	41	
40	Sahoo et al.^62^	India	Jamshedpur	6	2013–2014	Interview	Both	Hospital	Nonfatal	Mixed	101	42	59	
41	Saaiq and Ashraf^63^	Pakistan	Islamabad	24	2010–2012	Interviews and record review	Both	Hospital	Both	Burning	93	18	75	26.89 ± 6.1
42	Samaraweera et al.^64^	Sri Lanka	Ratnapura	3		Interviews, psychological autopsy	Urban	Community people	Fatal	Mixed	27	19	8	43
43	Shah et al.^65^	Bangladesh		6	2016–2017	Reviewing print news reports	Both	Community	Fatal	Mixed	271	113	158	26.67 ± 13.5
44	Sharmin Salam et al.^66^	Bangladesh	4 Subdistricts	6	2013	Interview	Rural	Community	Both	Mixed	95	48	47	
45	Srivastava^67^	India	Goa	36	2005–2007	Record review and interviews	Urban	Community	Fatal	Mixed	100	70	30	
46	Vijayakumar and Rajkumar^68^	India	Chennai	14	1994–1995	Interviews, and record review	Urban	Community	Fatal	Mixed	100	55	45	
47	Vijayakumar et al.^69^	India	Chennai	23	2002–2003	Interview	Urban	Hospital	Nonfatal		509	244	265	25.85 ± 9.3

### 3.2 Study quality assessment

As per modified Newcastle Ottawa Quality assessment scales for cross‐sectional study and case‐control study, 6 studies (*n* = 6, 12.8%) had high quality, 36 studies (*n* = 36, 76.6%) had moderate quality, and 5 studies (*n* = 5, 10.6%) had poor quality. Among 38 cross‐sectional studies, (1) the majority of the included studies' (34/38, 90%) sample were selected by nonrandom sampling methods, 7/38 (18%) studies used validated questionnaire tools, while 27/38 (71%) studies described the questionnaire tool although the validation was not clearly mentioned. Regarding the comparability of the different outcome groups, only 3/38 (8%) studies controlled for the important confounding factors. In the outcome assessments and statistical analysis domain, 22/38 (58%) studies collected self‐reported data, while the other studies used independent blind assessment and record linkage. Thirty‐two out of 38 (84%) studies clearly described the statistical tests (Supporting Information: File 2). Among the included nine case‐control studies, all the studies (9/9, 100%) clearly mention and applied the valid method for the selection of case, 8/9 (89%) studies selected community control, 7/9 (78%) studies controlled for the confounders. While the exposure was measured by semistructured interviews or psychological autopsy in all the studies (9/9, 100%) (Supporting Information: File 2).

### 3.3 Marital status in suicidal behavior

**Figure 2:**
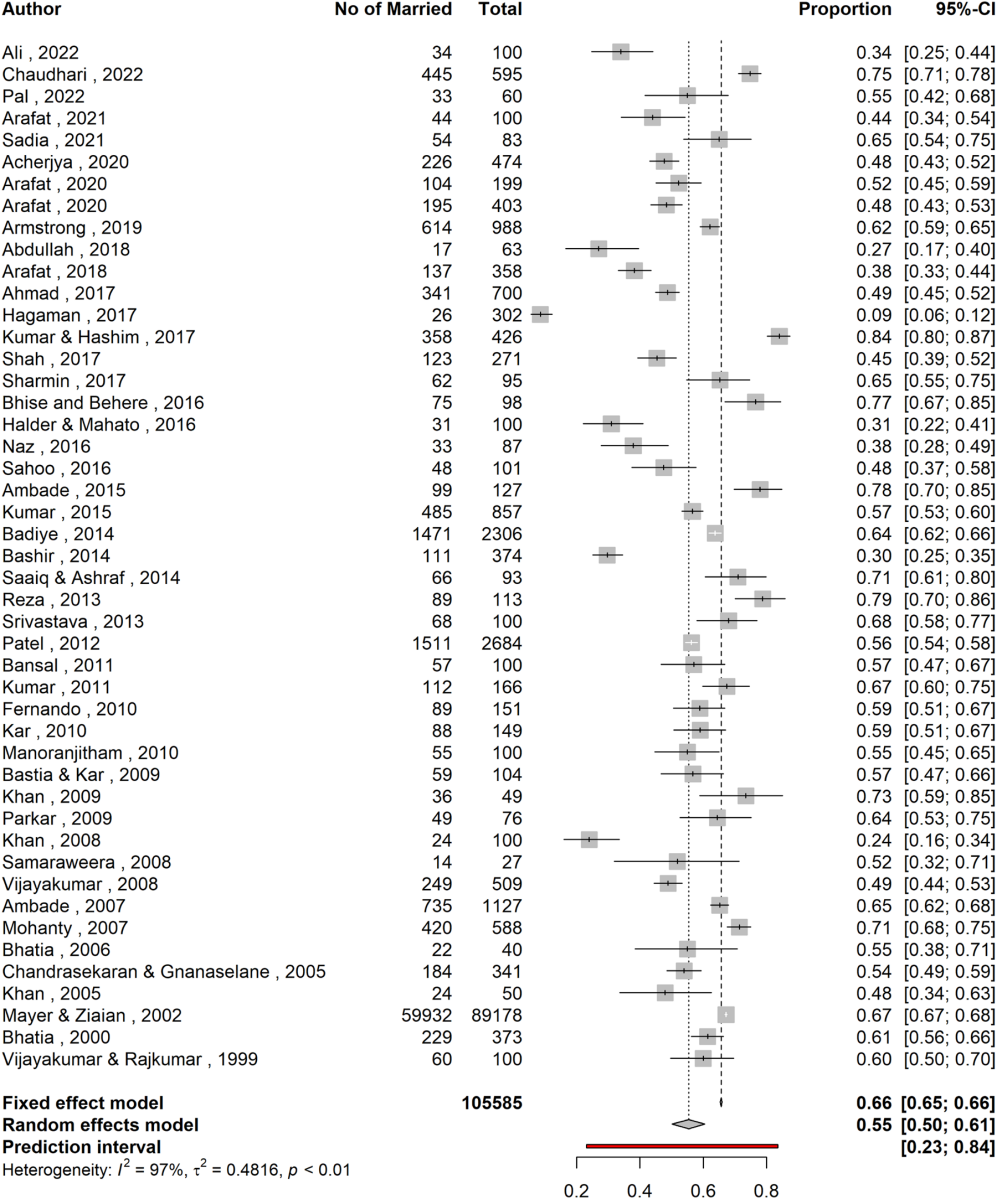
A forest plot showing the proportion of married individuals among all suicidal behavior.

**Figure 3:**
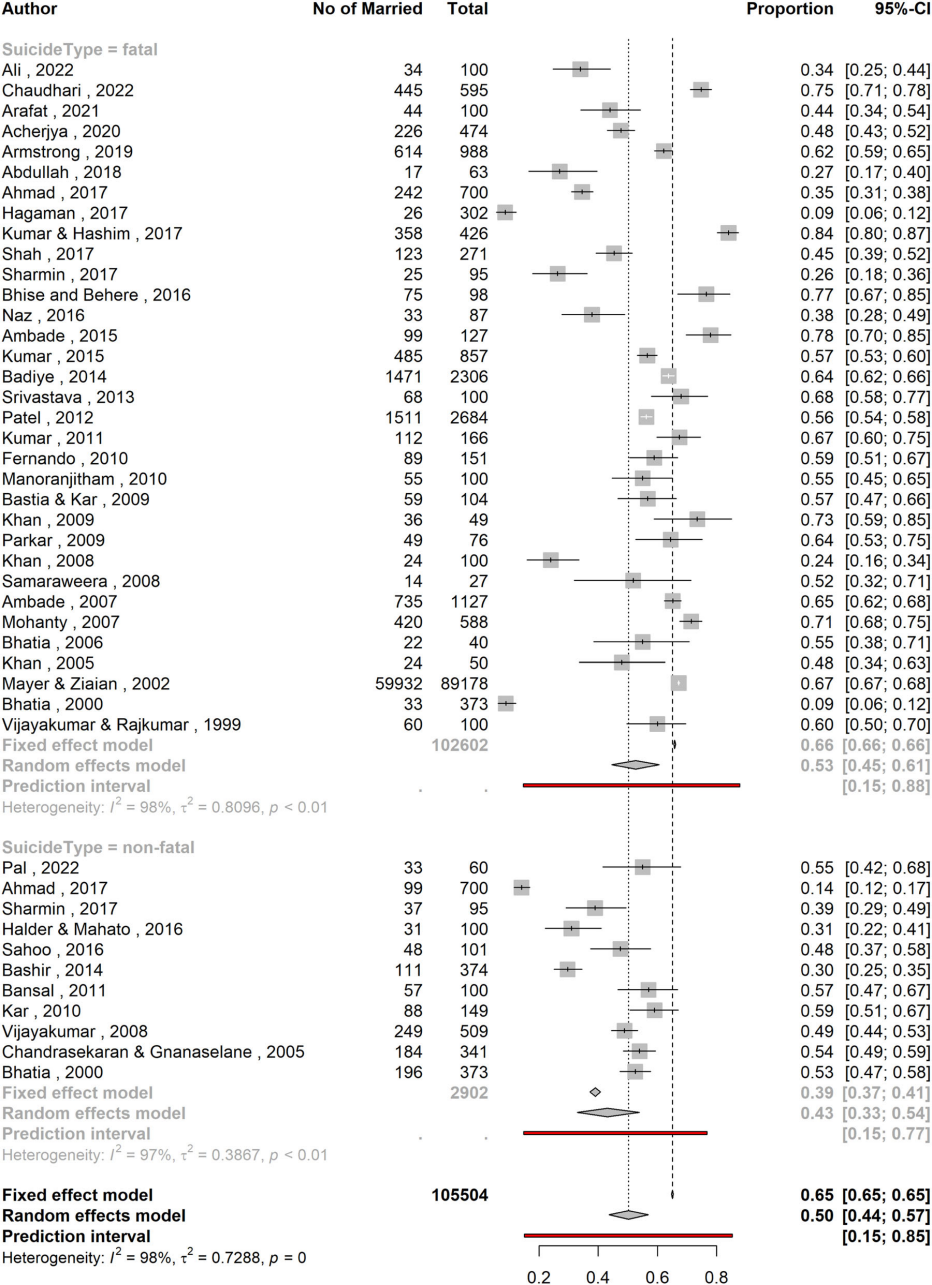
A forest plot showing the overall proportion of married individuals among suicide and suicide attempts.

The proportion of married individuals among persons with suicidal behavior was 55.4% (95% CI: 50.1–60.5; 47 studies; *n* = 105,585; *I* ^2^ = 96.9%, Figure 2). The studies were substantially heterogeneous (i.e., *I* ^2^ > 75% and *τ* ^2^ = 0.486), and the prediction interval of proportions ranged from 23.2% to 83.6%. The studies by Sadia et al.,^61^ Arafat et al.,^29^, ^30^, ^31^ Saaiq and Ashraf,^63^ and Reza et al.^60^ reported both fatal and nonfatal suicidal behavior but did not specify how many subjects had fatal and nonfatal behaviors. On the other hand, the studies by Ahmed et al.,^25^ Sharmin Salam et al.,^66^ and Bhatia et al.^39^ also reported both types of behaviors and specified their numbers. Hence, for subgroup analysis, between fatal and nonfatal suicide behavior the former six studies were excluded and the latter three studies were divided into two parts (fatal and nonfatal). The subgroup analysis (Figure 3) revealed that proportion of married individuals was 52.7% (95% CI: 44.6–60.7; 33 studies; *n* = 102,602; *I* ^2^ = 97.8%) in suicides and 43.1 (95% CI: 32.9*–*53.9; 11 studies; *n* = 2902; *I* ^2^ = 96.6%) in nonfatal attempts. Both these groups of studies had substantial heterogeneity (*I* ^2^ > 75%, *τ* ^2^ = 0.81 and 0.39). The prediction intervals were 14.7%–87.8% and 14.8%–76.8%, respectively. However, the difference was not significant (*p* = 0.13) (Table 2).

**Table 2:** Statistical comparison of pooled proportions of married individuals with suicidal behavior across different subgroups.

Subgroups	Pooled proportions	95% CI	*I* ^2^	*p*_subgroup_
Outcome				0.13
Suicide (*k* = 33)	0.53	0.45–0.61	97.8%	
Nonfatal attempt (*k* = 11)	0.43	0.33*–*0.54	96.6%	
Country				**0.015**
Pakistan (*k* = 9)	0.45	0.31*–*0.59	93.2%	
Bangladesh (*k* = 8)	0.52	0.42*–*0.63	89.0%	
India (*k* = 27)	0.62	0.57*–*0.66	94.6%	
Quality				0.63
Low (*k* = 5)	0.54	0.38– 0.69	83.5%	
Moderate (*k* = 36)	0.56	0.38*–*0.69	96.9%	
High (*k* = 6)	0.51	0.35*–*0.65	91.4%	

*Note*: Bold indicates *p* < 0.05.

### 3.4 Country‐wise variation

**Figure 4:**
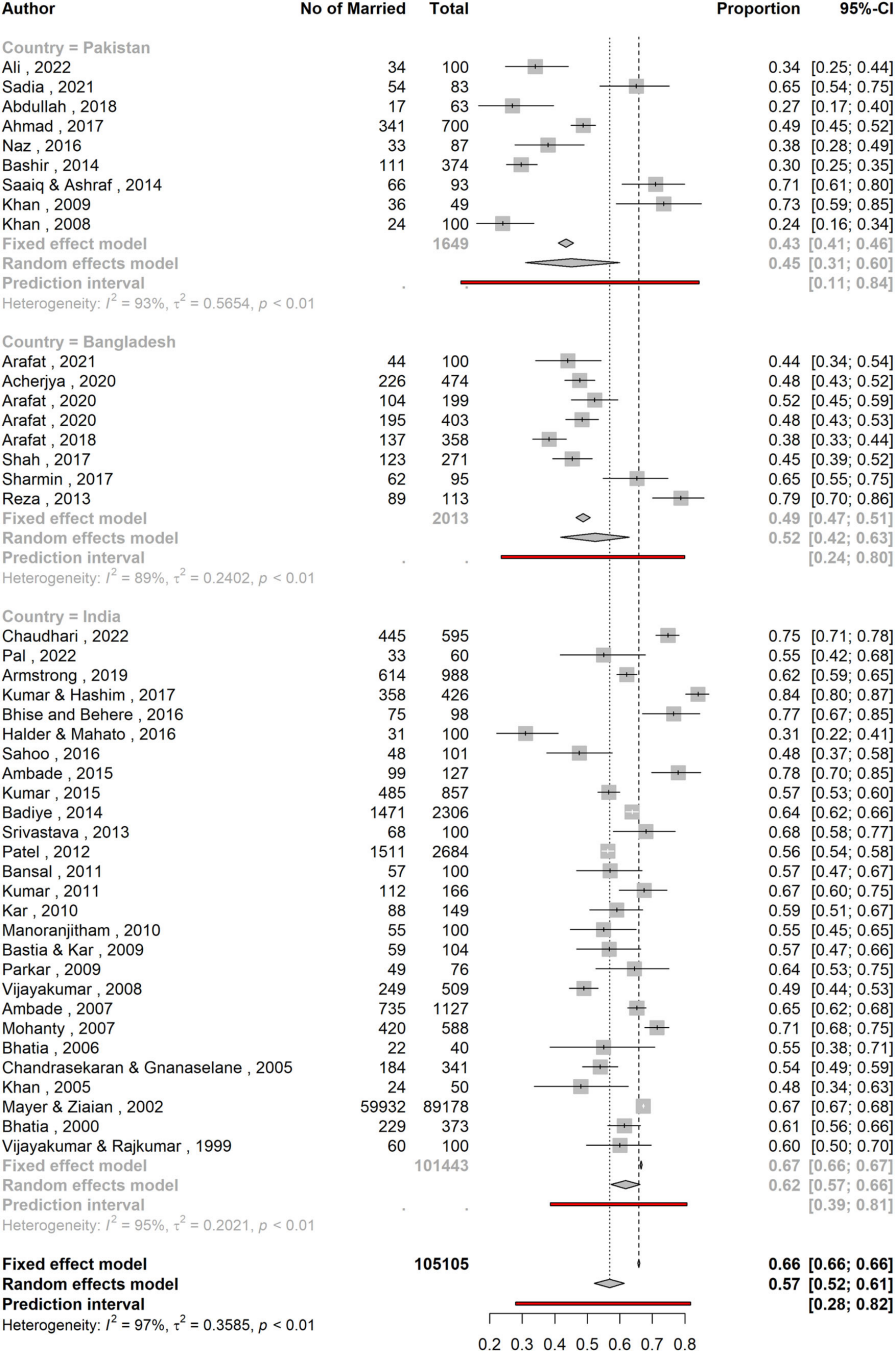
A forest plot showing proportion of married individuals with suicidal behavior across countries.

The proportion of married persons in attempted suicide cases varied significantly across countries (*p* = 0.016, Table 2). Studies in India found the highest proportion (61.8%; 95% CI: 57.2*–*66.2; *n* = 101,443) followed by Bangladesh (52.5%; 95% CI: 41.8%–62.9%; *n* = 2013) and Pakistan (45.1%; 95% CI: 30.9–59.9; *n* = 1649). However, studies from Bangladesh were relatively less heterogeneous (*I* ^2^ = 89.0%, *τ* ^2^ = 0.240) compared to those of India (*I* ^2^ = 94.6%, *τ* ^2^ = 0.202) and Pakistan (*I* ^2^ = 93.2%, *τ* ^2^ = 0.565). Nonetheless, all of these studies had substantial heterogeneity (*I* ^2^ > 75%). Prediction intervals were 38.6%–80.6% for India, 23.5%–79.9% for Bangladesh, and 11.1%–84.3% for Pakistan (Figure 4).

**Figure 5:**
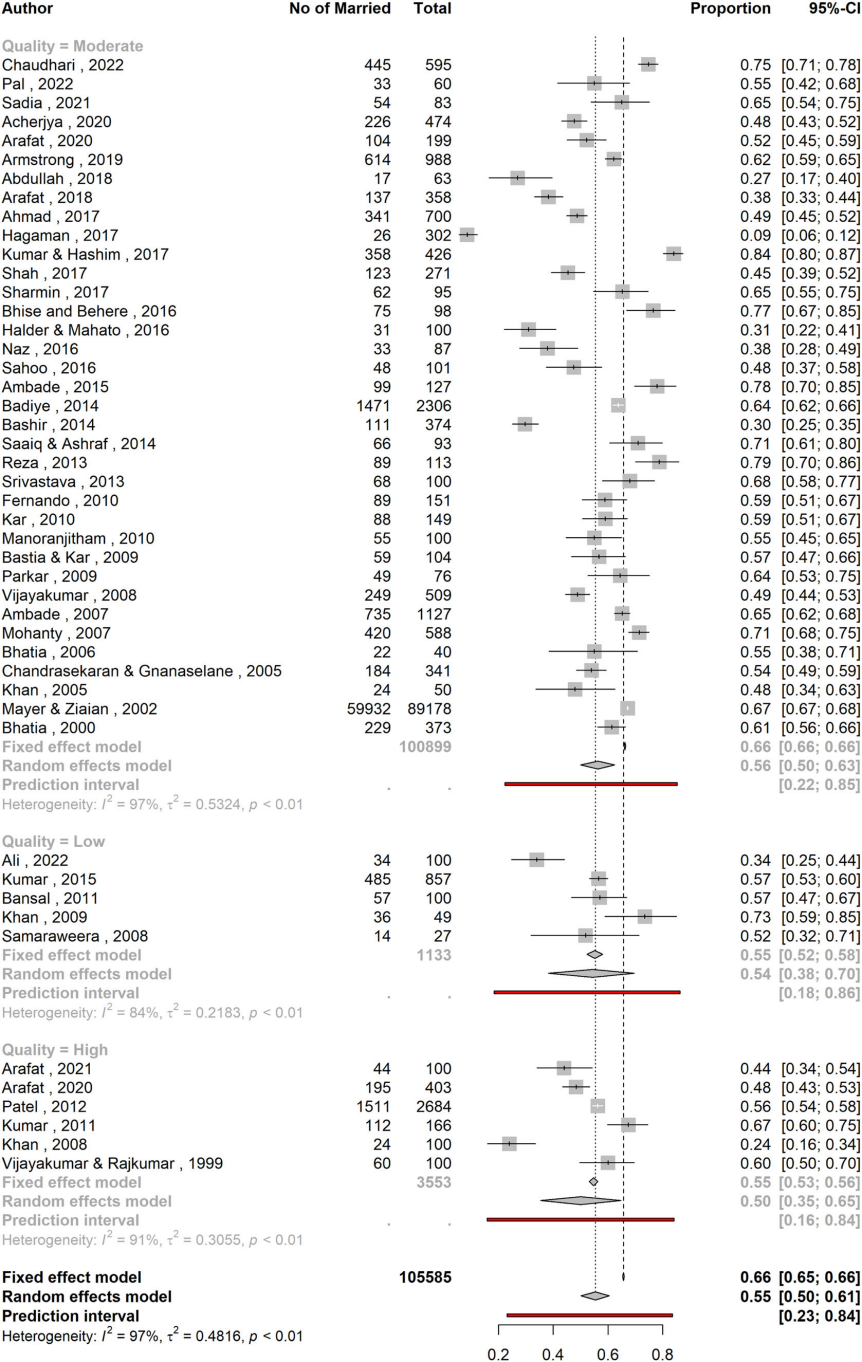
A forest plot showing the proportion of married individuals with suicidal behavior stratified by study quality.

The pooled proportions did not differ significantly in relation to the quality of the studies (*p* = 0.63, Table 2). The proportion estimates were 54.4% (95% CI: 38.3–69.7; 5 studies; *n* = 1133) for low‐quality studies, 56.4% (95% CI: 50.1–62.5; 36 studies; *n* = 100,899) for medium‐quality studies, and 50.1% (95% CI: 35.4–64.8; 6 studies; *n* = 3553) for high‐quality studies. The medium‐quality studies had the highest heterogeneity (*I* ^2^ = 96.9%, *τ* ^2^ = 0.532), and low‐quality studies had the lowest heterogeneity (*I* ^2^ = 83.5%, *τ* ^2^ = 0.218). The high‐quality studies had moderate heterogeneity (*I* ^2^ = 97%, *τ* ^2^ = 0.305). The prediction intervals were 18.2%–86.3%, 22.3%–85.3%, and 15.9%–84.2%, respectively (Figure 5).

## 4 DISCUSSION

### 4.1 Major findings of the study

The aim of this systematic review was to determine the marital status in individuals with suicidal behavior (fatal, nonfatal, or both) in South Asian countries. By analyzing a total of 47 studies, we found several key findings that shed light on this relationship between marital status and suicidal behavior in the region. Our analysis revealed that the proportion of married individuals among persons with suicidal behavior in South Asia was 55.4%. This finding suggests that marital status may play a significant role in suicidal behavior in this region. However, it is important to note the high heterogeneity among studies included in our review. This indicates that there is considerable variability in the estimates across studies, which may be attributed to differences in sample characteristics, study designs, and measurement instruments.

When examining the specific types of suicidal behavior, our subgroup analysis showed that the proportion of married individuals among suicides was 52.7%, while among nonfatal suicide attempts it was 43.1%. Although the difference between these two groups was not statistically significant, these findings suggest that marital status may have varying degrees of association with different forms of suicidal behavior. Further research is needed to explore this association in more depth and investigate potential underlying factors.

Our analysis did not find a significant difference in the proportion of married individuals among persons with suicidal behavior based on the quality of the studies. This suggests that the association between marital status and suicidal behavior is consistent across studies with varying methodological quality. However, it is worth noting that the majority of the included studies were of moderate or poor quality, indicating the need for more rigorous research in this area.

### 4.2 Implications of the study results

Our findings have two important implications. First, the relationship between marital status and suicidal behavior in South Asia appears to exhibit unique patterns compared to findings elsewhere. In many Western countries, being unmarried or divorced is often associated with a higher risk of suicidal behavior, while being married is generally considered protective.^4^, ^70^ However, studies in South Asia have shown a higher proportion of married individuals among those engaging in suicidal behavior.^59^, ^68^, ^71^, ^72^, ^73^ This contrasting finding suggests that the association between marital status and suicidal behavior may be influenced by cultural, social, and economic factors specific to the South Asian region. Specifically, gender stereotyping, limited agency for women, and the expectation of fulfilling certain marital responsibilities may contribute to stress and psychological distress within marriages, potentially increasing the risk of suicidal behavior among married individuals, particularly among women.^59^, ^68^ In South Asian countries, females have low economic freedom and mostly they are engaged in household activities where a good proportion is living with in‐laws. Many times, females attempt suicide due to conflicts among family members. Additionally, before marriage, females get supportive reactions from parents even in irrational activities which changes in the in‐laws' environment. Further studies focusing on immediate events of suicidal behavior are warranted to assess the complexity as well as to formulate culture sensitive interventions for suicide prevention. Based on speculations, family members could be potential persons for preventing suicide attempts. Several measures have been proposed for family members like creating awareness about life‐events and suicide attempts, gate‐keeper training, supportive and healthy communications among the family members, and talking to the marital therapist.^74^ However, empirical studies are warranted to validate the speculations.

Secondly, we also observed significant country‐wise variation in the proportion of married individuals among attempted suicide cases. Studies conducted in India reported the highest proportion (61.8%), followed by Bangladesh (52.5%) and Pakistan (45.1%). These findings indicate that cultural and social factors may moderate the association between marital status and suicidal behavior in South Asian countries. Context‐specific factors such as religion, gender roles, societal norms, and marital expectations, which may differ between settings, could contribute to these variations. Among these three countries, India is a Hindu‐majority country whereas Bangladesh and Pakistan are Muslim‐majority countries. The suicide rate is higher in India when compared to Bangladesh and Pakistan.^1^ Therefore, religion may be an attributing factor aligning the total rate of suicide. Another possible factor could be the use of alcohol which is higher in India than in Bangladesh and Pakistan.

### 4.3 Strength and limitations

To the best of the authors' knowledge, this is the first study assessing the marital status in suicidal behavior in South Asia. However, the present systematic review had some key limitations. First, the analysis may not reflect marital status as a risk factor as these findings may reflect the proportion of married persons in the community. Second, the high heterogeneity among the included studies in terms of study design, populations, and measurement tools may have influenced pooled estimates and may affect the generalizability of results. Third, the potential for publication bias was not assessed due to the nature of studies included in this review. Fourth, the reliance on self‐reported data in some studies may introduce biases and affect the accuracy of the estimates. Fifth, because we included only studies done on patients with suicidal behavior, we were unable to estimate associations between different types of marital status and suicidal behavior in the region. Sixth, the age distribution of married persons may vary across the countries in the world. In many nations' populations, the married population is large and often in a majority in the fifth–sixth decade of their which is different in South Asian countries. Additionally, we did not compare the estimates to the national proportions of the population. Seventh, we were unable to perform the risk ratio for different marital statuses. Because, the necessary data to perform the calculation were not available in the articles. Additionally, the available data were not collected from nationwide studies and official estimates of suicidal behavior were not compared. Eighth, we did not perform the effect of age and gender due to unavailability of necessary data.

## 5 CONCLUSIONS

This systematic review provides insights into the association between marital status and suicidal behavior in South Asia. This review identified married persons died more than others by suicide in South Asian countries while single persons attempted suicide than married. As the current study did not assess any cause‐and‐effect association, a cautious interpretation is warranted while considering married marital status as a risk factor. The findings suggest that marital status may play a role in suicidal behavior, but further research is needed to better understand the underlying mechanisms and contextual factors which is necessary to formulate and implement culture‐sensitive suicide prevention strategies. Future studies should consider employing standardized methodologies and addressing the limitations identified in this review to enhance the robustness of the evidence. Understanding the association between marital status and suicidal behavior can inform the development of targeted interventions and support strategies aimed at reducing suicide rates in South Asia.

## AUTHOR CONTRIBUTIONS

**S. M. Yasir Arafat**: Conceptualization; data curation; investigation; project administration; writing—original draft; writing—review & editing. **Vikas Menon**: Formal analysis; writing—original draft; writing—review & editing. **Md Abdullah Saeed Khan**: Formal analysis; writing—original draft; writing—review & editing. **Mila Nu Nu Htay**: Methodology; writing—original draft; writing—review & editing. **Rakesh Singh**: Writing—original draft; writing—review & editing. **Deepika Biyyala**: Methodology; writing—original draft; writing—review & editing. **Yuvaraj Krishnamoorthy**: Formal analysis. **Keerthana Mynampally**: Methodology.

## CONFLICT OF INTEREST STATEMENT

S. M. Yasir Arafat is an editorial board member. The remaining authors declare no conflict of interest.

## TRANSPARENCY STATEMENT

The lead author S. M. Yasir Arafat affirms that this manuscript is an honest, accurate, and transparent account of the study being reported; that no important aspects of the study have been omitted; and that any discrepancies from the study as planned (and, if relevant, registered) have been explained.

## Supporting information

Supporting information.

Supporting information.

## Data Availability

The data that support the findings of this study are available on request from the corresponding author.
