# Supplementary material for: Marital status and suicidal behavior in South Asia: A systematic review and meta‐analysis
Source: Health Sci Rep. 2023 Dec 20;6(12):e1781. doi: 10.1002/hsr2.1781 (PMC10733573; doi:10.1002/hsr2.1781)
Supplement: Supplementary file 1 — Supporting information. [file HSR2-6-e1781-s002.docx]

Supplementary file 1: Search details

For our searches of the databases, we used the following search terms:

((mari*) or (marital and status)) OR ((mari*) or (marital and status)).ti

AND

(self?harm* or suicid*).ab OR (self?harm* or suicid*).ti

AND

(Afghanistan* or Bangladesh* or Bhutan* or India* or Maldiv* or Nepal* or Pakistan* or Sri Lanka*).ab OR (Afghanistan* or Bangladesh* or Bhutan* or India* or Maldiv* or Nepal* or Pakistan* or Sri Lanka*).ti

Databases: MEDLINE, EMBASE, and PsycINFO

Date range: from inception to the search date

Search date: February 04, 2023 at 12.00 PM (Dhaka time)
