# Supplementary material for: Marital status and suicidal behavior in South Asia: A systematic review and meta‐analysis
Source: Health Sci Rep. 2023 Dec 20;6(12):e1781. doi: 10.1002/hsr2.1781 (PMC10733573; doi:10.1002/hsr2.1781)
Supplement: Supplementary file 2 — Supporting information. [file HSR2-6-e1781-s001.docx]

Supplementary file 2: Quality assessment

Appendix table 1. Quality assessment of included cross-sectional studies (n=38)

| **SN** | **Study** | **Selection** | | | | | | **Comparability** | | **Outcome** | | | | Total Score | Quality |
| --- | --- | --- | --- | --- | --- | --- | --- | --- | --- | --- | --- | --- | --- | --- | --- |
|  |  | Representativeness of the sample | | Sample size | Non-respondents | exposure | | Different outcome groups are comparable, Confounding factors are controlled | | Assessment | | | Statistical test |  |  |
|  |  | *Truly representative (all subjects or random sampling) | *Somewhat representative (Non random sampling) | *Justify | *Comparability R and NR | **Validated measurement tool. | *NV but tool is available or described | *Controls for the most important factor | *control for any additional factor | **Independent blind assessment | **Record linkage | *Self report | *clearly described and appropriate |  |  |
| 1 | Abdullah et al., 2018 | 0 | 1 | 0 | 0 | 0 | 1 | 0 | 0 | 0 | 2 | 0 | 1 | 5 | Moderate |
| 2 | Acherjya et al., 2020 | 0 | 1 | 0 | 0 | 0 | 1 | 0 | 0 | 0 | 0 | 1 | 1 | 4 | Moderate |
| 3 | Ahmad et al., 2017 | 0 | 1 | 0 | 0 | 0 | 0 | 0 | 0 | 0 | 2 | 1 | 1 | 5 | Moderate |
| 4 | Ambade et al., 2007 | 0 | 1 | 0 | 0 | 0 | 1 | 0 | 0 | 0 | 2 | 0 | 1 | 5 | Moderate |
| 5 | Ambade et al., 2015 | 0 | 1 | 0 | 0 | 0 | 1 | 0 | 0 | 0 | 2 | 0 | 1 | 5 | Moderate |
| 6 | Arafat et al., 2020a | 0 | 1 | 0 | 0 | 0 | 1 | 0 | 0 | 0 | 2 | 0 | 1 | 5 | Moderate |
| 7 | Arafat et al., 2020b | 0 | 1 | 0 | 0 | 0 | 1 | 0 | 0 | 2 | 2 | 0 | 1 | 7 | High |
| 8 | Arafat et al., 2018 | 0 | 1 | 0 | 0 | 0 | 1 | 0 | 0 | 0 | 2 | 0 | 1 | 5 | Moderate |
| 9 | Armstrong et al., 2019 | 0 | 1 | 0 | 0 | 0 | 1 | 0 | 0 | 0 | 2 | 0 | 1 | 5 | Moderate |
| 10 | Badiye et al., 2014 | 0 | 1 | 0 | 0 | 0 | 1 | 0 | 0 | 0 | 2 | 0 | 1 | 5 | Moderate |
| 11 | Bansal et al., 2011 | 0 | 1 | 0 | 0 | 0 | 0 | 0 | 0 | 0 | 0 | 1 | 1 | 3 | Low |
| 12 | Bashir et al., 2014 | 0 | 1 | 0 | 0 | 2 | 0 | 0 | 0 | 0 | 0 | 1 | 1 | 5 | Moderate |
| 13 | Bastia & Kar, 2009 | 0 | 1 | 0 | 0 | 0 | 1 | 0 | 0 | 0 | 2 | 1 | 0 | 5 | Moderate |
| 14 | Bhatia et al., 2006 | 0 | 1 | 0 | 0 | 0 | 1 | 0 | 0 | 0 | 2 | 1 | 1 | 6 | Moderate |
| 15 | Bhatia et al., 2000 | 0 | 1 | 0 | 0 | 2 | 0 | 0 | 0 | 0 | 0 | 1 | 0 | 4 | Moderate |
| 16 | Chandrasekaran & Gnanaselane, 2005 | 0 | 1 | 0 | 0 | 2 | 0 | 0 | 0 | 0 | 0 | 1 | 1 | 5 | Moderate |
| 17 | Chaudhari et al., 2022 | 0 | 1 | 0 | 0 | 0 | 1 | 0 | 0 | 0 | 2 | 1 | 1 | 6 | Moderate |
| 18 | Fernando et al., 2010 | 0 | 1 | 0 | 0 | 0 | 1 | 0 | 0 | 0 | 0 | 1 | 1 | 4 | Moderate |
| 19 | Hagaman et al., 2017 | 0 | 1 | 0 | 0 | 0 | 1 | 0 | 0 | 0 | 0 | 1 | 1 | 4 | Moderate |
| 20 | Halder & Mahato, 2016 | 0 | 1 | 0 | 0 | 2 | 0 | 0 | 0 | 0 | 0 | 1 | 0 | 4 | Moderate |
| 21 | Kar, 2010 | 0 | 1 | 0 | 0 | 2 | 0 | 0 | 0 | 0 | 0 | 1 | 1 | 5 | Moderate |
| 22 | Khan et al., 2005 | 0 | 1 | 0 | 0 | 0 | 1 | 0 | 0 | 0 | 0 | 1 | 1 | 4 | Moderate |
| 23 | Khan et al., 2009 | 0 | 1 | 0 | 0 | 0 | 0 | 0 | 0 | 0 | 2 | 0 | 0 | 3 | Low |
| 24 | Kumar et al., 2015 | 0 | 1 | 0 | 0 | 0 | 0 | 0 | 0 | 0 | 2 | 0 | 0 | 3 | Low |
| 25 | Kumar & Hashim, 2017 | 0 | 1 | 0 | 0 | 0 | 1 | 0 | 0 | 0 | 2 | 0 | 1 | 5 | Moderate |
| 26 | Mayer & Ziaian, 2002 | 1 | 0 | 0 | 0 | 0 | 1 | 0 | 0 | 0 | 2 | 0 | 1 | 5 | Moderate |
| 27 | Mohanty et al., 2007 | 0 | 1 | 0 | 0 | 0 | 1 | 1 | 0 | 0 | 2 | 0 | 1 | 6 | Moderate |
| 28 | Naz, 2016 | 0 | 1 | 0 | 0 | 0 | 1 | 0 | 0 | 0 | 2 | 0 | 1 | 5 | Moderate |
| 29 | Parkar et al., 2009 | 0 | 1 | 0 | 0 | 0 | 1 | 0 | 0 | 0 | 0 | 1 | 1 | 4 | Moderate |
| 30 | Patel et al., 2012 | 1 | 0 | 0 | 0 | 0 | 1 | 1 | 1 | 0 | 2 | 1 | 1 | 8 | High |
| 31 | Sadia et al., 2021 | 0 | 1 | 0 | 0 | 0 | 1 | 0 | 0 | 0 | 2 | 0 | 1 | 5 | Moderate |
| 32 | Sahoo et al., 2016 | 0 | 1 | 0 | 0 | 2 | 0 | 0 | 0 | 0 | 0 | 1 | 1 | 5 | Moderate |
| 33 | Saaiq & Ashraf, 2014 | 0 | 1 | 0 | 0 | 0 | 1 | 0 | 0 | 0 | 2 | 1 | 1 | 6 | Moderate |
| 34 | Samaraweera et al., 2008 | 0 | 1 | 0 | 0 | 0 | 1 | 0 | 0 | 0 | 0 | 1 | 0 | 3 | Low |
| 35 | Shah et al., 2017 | 1 | 0 | 0 | 0 | 0 | 1 | 0 | 0 | 2 | 0 | 0 | 1 | 5 | Moderate |
| 36 | Sharmin Salam et al., 2017 | 1 | 0 | 0 | 0 | 0 | 1 | 1 | 1 | 0 | 0 | 1 | 1 | 6 | Moderate |
| 37 | Srivastava, 2013 | 0 | 1 | 0 | 0 | 0 | 1 | 0 | 0 | 0 | 0 | 1 | 1 | 4 | Moderate |
| 38 | Vijayakumar et al., 2008 | 0 | 1 | 0 | 0 | 2 | 0 | 0 | 0 | 0 | 0 | 1 | 1 | 5 | Moderate |

Appendix table 2. Quality assessment of included case control studies (n=9)

| **SN** | **Author** | **Selection** | | | | | | | | | | **Comparability** | | **Exposure** | | | | | | | | |  |
| --- | --- | --- | --- | --- | --- | --- | --- | --- | --- | --- | --- | --- | --- | --- | --- | --- | --- | --- | --- | --- | --- | --- | --- |
|  |  | **Case definition** | | | **Representativeness of cases** | | **Selection of controls** | | | **Definition of controls** | |  |  | **Ascertainment of Exposure** | | | **Same method of ascertainment for cases and controls** | | **Non-response rate** | | |  | Quality |
|  |  | Yes, with independent validation (*) | Yes, record linkage or self reports | No description | Representative series of cases (*) | Selection bias or not stated | Community control (*) | Hospital control | No description | No history of disease (endpoint) (*) | No description | Control for confonder (*) | Control for additional factor (*) | Secure records (Surgical records) (*) | Structured interview-blind (*) | Not blinded, Self report or medical records only or no description | Yes (*) | No | Same rate in both groups (*) | non respondents described | rate different and no designation | Total score |  |
| 1 | Ali et al., 2022 | 1 | 0 | 0 | 0 | 0 | 1 | 0 | 0 | 0 | 0 | 0 | 0 | 0 | 0 | 0 | 1 | 0 | 0 | 0 | 0 | 3 | Low |
| 2 | Arafat et al., 2021b | 1 | 0 | 0 | 1 | 0 | 1 | 0 | 0 | 0 | 0 | 1 | 1 | 0 | 0 | 0 | 1 | 0 | 1 | 0 | 0 | 7 | High |
| 3 | Bhise and Behere, 2016 | 1 | 0 | 0 | 1 | 0 | 1 | 0 | 0 | 1 | 0 | 1 | 0 | 0 | 0 | 0 | 1 | 0 | 0 | 0 | 0 | 6 | Moderate |
| 4 | Khan et al., 2008 | 1 | 0 | 0 | 1 | 0 | 1 | 0 | 0 | 1 | 0 | 1 | 1 | 0 | 0 | 0 | 1 | 0 | 0 | 0 | 0 | 7 | High |
| 5 | Kumar et al., 2011 | 1 | 0 | 0 | 1 | 0 | 1 | 0 | 0 | 1 | 0 | 1 | 1 | 0 | 0 | 0 | 1 | 0 | 0 | 0 | 0 | 7 | High |
| 6 | Manoranjitham et al., 2010 | 1 | 0 | 0 | 1 | 0 | 1 | 0 | 0 | 1 | 0 | 1 | 0 | 0 | 0 | 0 | 1 | 0 | 0 | 0 | 0 | 6 | Moderate |
| 7 | Pal et al., 2022 | 1 | 0 | 0 | 1 | 0 | 0 | 0 | 0 | 1 | 0 | 0 | 0 | 0 | 0 | 0 | 1 | 0 | 0 | 0 | 0 | 4 | Moderate |
| 8 | Reza et al., 2013 | 1 | 0 | 0 | 1 | 0 | 1 | 0 | 0 | 1 | 0 | 1 | 0 | 0 | 0 | 0 | 1 | 0 | 0 | 0 | 0 | 6 | Moderate |
| 9 | Vijayakumar & Rajkumar, 1999 | 1 | 0 | 0 | 1 | 0 | 1 | 0 | 0 | 1 | 0 | 1 | 1 | 0 | 0 | 0 | 1 | 0 | 0 | 0 | 0 | 7 | High |
